# Duplex High-Resolution Melting Assay for the Simultaneous Genotyping of IL28B rs12979860 and PNPLA3 rs738409 Polymorphisms in Chronic Hepatitis C Patients

**DOI:** 10.3390/ijms160922223

**Published:** 2015-09-14

**Authors:** Elena L. Enache, Anca Sin, Ligia Bancu, Christophe Ramière, Olivier Diaz, Patrice André, Liviu S. Enache

**Affiliations:** 1 University of Medicine and Pharmacy Tirgu Mures, 38 Gh. Marinescu st., Tirgu Mures 540142, Romania; E-Mails: dincaluminita@yahoo.com (E.L.E.); anka_sinn@yahoo.com (A.S.); enachesliviu@yahoo.com (L.S.E.); 2 Emergency County Clinical Hospital, 50 Gh. Marinescu st., Tirgu Mures 540136, Romania; 3 Université de Lyon, Université Lyon 1, Lyon F-69008, France; E-Mails: christophe.ramiere@inserm.fr (C.R.); olivier.diaz@inserm.fr (O.D.); patrice.andre@inserm.fr (P.A.); 4 Inserm U1111, 21 Avenue Tony Garnier, Lyon F-69007, France; 5 CIRI, Centre International de Recherche en Infectiologie, Université de Lyon, 21 Avenue Tony Garnier, 69365 Lyon Cedex 07, France; 6 Ecole Normale Supérieure de Lyon, 15 parvis René Descartes, BP 7000 69342 Lyon Cedex 07, France; 7 CNRS, UMR5308, 21 avenue Tony Garnier, 69365 Lyon Cedex 07, France; 8 Hospices Civils de Lyon, Hôpital de la Croix Rousse, Laboratoire de Virologie, Lyon F-69004, France

**Keywords:** chronic hepatitis C, personalized diagnostics, duplex assay, high-resolution melting, interleukin 28B, patatin-like phospholipase domain containing 3 (PNPLA3), single nucleotide polymorphism

## Abstract

Chronic hepatitis C (CHC) is a major burden for public health worldwide. Although newer direct-acting antivirals show good efficacy, their cost precludes their wide adoption in resource-limited regions. Thus, strategies are being developed to help identify patients with high susceptibility to response to classic PEG-interferon + ribavirin therapy. *IL28B* polymorphism rs12979860 C/T is an important predictor for an efficient response to interferon-based therapy. A genetic variant in adiponutrin (*PNPLA3*) gene, rs738409 C/G, is associated with steatosis, severity, and progression of liver fibrosis in CHC patients, and predicts treatment outcome in difficult-to-cure HCV-infected patients with advanced fibrosis. We developed a rapid and inexpensive assay based on duplex high-resolution melting (HRM) for the simultaneous genotyping of these two polymorphisms. The assay validation was performed on synthetic DNA templates and 132 clinical samples from CHC patients. When compared with allele-specific PCR and sequencing, our assay showed 100% (95% CI: 0.9724–1) accuracy, with 100% sensitivity and specificity. Our assay was robust against concentration and quality of DNA samples, melting curve normalization intervals, HRM analysis algorithm, and sequence variations near the targeted SNPs (single nucleotide polymorphism). This duplex assay should provide useful information for patient-oriented management and clinical decision-making in CHC.

## 1. Introduction

Hepatitis C virus (HCV) infection is a major cause of liver disease, with approximately 80 million viremic infections worldwide [1]. Epidemiological data suggests that HCV infection may be “more deadly” than previously considered [1]. Up to 80% of the infected patients become chronic carriers [2] and 20% of chronic hepatitis C (CHC) lead to cirrhosis. CHC remains the leading cause of end-stage liver disease and hepatocellular carcinoma in Western countries [3]. Hepatic steatosis is a central pathological feature of CHC. It occurs in about 40% of HCV infected patients, independently of other risk factors of fatty liver [4]. It is associated with advanced fibrosis and poorer response to interferon (IFN)-based treatment [5]. Recent studies revealed several host genetic factors predictive for HCV disease progression and treatment outcome.

Until 2011, the standard treatment of HCV infection consisted of a combination of PEG-interferon (PEG-IFN) and ribavirin (RBV), with less than 50% of patients infected with HCV genotype 1 achieving SVR; *i.e.*, viral clearance [6]. The first developed inhibitors of HCV NS3/4A protease (e.g., telaprevir, boceprevir) showed promising results in combination with PEG-IFN/RBV, and clinical trials reported success rates of more than 70% in patients treated with triple therapy [7]. Unfortunately, antiviral treatment in CHC did not have any significant impact on the number of infections worldwide [1]. The future second-generation of HCV NS3/4A protease inhibitor, HCVNS5A inhibitor, and HCV NS5B inhibitor treatments, despite their high efficacy and a lower toxicity, were singled out as expensive. High costs of therapies and diagnostics have been cited among factors contributing to low treatment rates and success, especially in low-resources regions [8].

It is estimated that, for the next years, CHC treatment will still rely on PEG-IFN/RBV and triple therapy in the majority of the world’s areas, until the newest direct-acting antivirals become approved or reimbursed [9]. Due to the substantial increase in therapy costs implied by the latest medications, but also to IFN and protease inhibitors related side effects and tolerance issues, strategies are being developed to help identify patients with high chances of response to classic therapy [9].

A non-synonymous variant (rs738409) of the patatin-like phospholipase domain containing 3 (*PNPLA3*) gene was first identified to be associated with liver fat content and risk of non-alcoholic fatty liver disease in individuals from various ancestries [10]. Subsequent investigations linked allele G of this site to the presence of steatosis, intense necroinflammatory activity, and advanced fibrosis in CHC patients [11,12,13,14,15]. *PNPLA3* genotype GG is also a negative predictor for antiviral treatment outcome in difficult-to-cure patients with HCV genotype 1 or 4 infection and advanced fibrosis (sustained viral response (SVR) achieved in 17% *vs.* 46% of patients) [16]. The role of this polymorphism in liver pathology is further suggested by its association with increased risk of non-alcoholic steatohepatitis [17], liver cirrhosis [18], and hepatocellular carcinoma in patients with underlying liver disease [19,20].

Several single nucleotide polymorphisms (SNP) in the interleukin-28B (*IL28B*) region are associated with the outcome of PEG-IFN/RBV therapy in CHC patients [21,22,23]. Individuals with a favorable genotype have a probability of achieving SVR at least twice as high as those with an unfavorable genotype [24]. The same polymorphisms also remain valuable in the prediction of triple therapy success [9,25,26]. Among these, the most studied SNP is rs12979860 [24]. Genetic variation in the *IL28B* region also seems to be associated with HCV-induced liver inflammation, fibrosis, and steatosis [27,28,29]. At present, *IL28B* genotyping is recommended in cases where only PEG-IFN/RBV treatment is available or acceptable, or to aid in selecting cost-effective treatment options in resource-limited settings [30].

In order to improve accessibility to adapted treatments using affordable diagnostic methods, we have developed and characterized a rapid and inexpensive assay based on duplex high-resolution melting (HRM) analysis. This strategy allows the simultaneous genotyping of rs12979860 and rs738409, two independent factors associated with disease progression and response to treatment in CHC.

## 2. Results

### 2.1. Primer Characterization in Singleplex Reactions

HRM primers were first validated in singleplex reactions using a PikoReal 96 instrument (Thermo Fisher Scientific, Vantaa, Finland). Primer specificity was assessed by agarose electrophoresis of PCR products, which revealed single bands of the expected sizes. Using serial dilutions of standard templates for each genotype in the range of 1.8 × 10^6^ to 1.8 × 10^1^ copies/reaction, we calculated the amplification efficiencies of singleplex HRM reactions to be 96.4% for rs12979860 and 92.1% for rs738409. Linearity was maintained and the correct call was obtained for all genotypes throughout the tested template concentrations.

Singleplex HRM reactions correctly identified genotypes of all 132 clinical samples tested. Results were confirmed by allele-specific real-time PCR. Sanger sequencing was available for 123 samples (rs12979860: 32 CC, 69 CT, 22 TT; rs738409: 68 CC, 51 CG, four GG) and was concordant with real-time PCR assays. Genotype distributions (Table 1) for both SNPs were in Hardy-Weinberg equilibrium (*p* > 0.05, Chi-squared test). As expected, the two SNPs were in linkage disequilibrium (Dʹ = 0.3, *R*^2^ = 0.04, *p* > 0.05). The diplotype frequencies encountered in our patient cohort (Table 2) are in concordance with previous studies on Caucasian population [12,31]. IL28B rs12979860 CC genotype was significantly associated with SVR rate (*p* = 0.02), whereas TT was predictive for severe necroinflammation (A3, *p* = 0.03). Due to the limited sample size, we did not observe an association between *PNPLA3* genotype and advanced fibrosis (F3–F4) in our patients.

**Table 1:** Demographic, biological, and virological characteristics of patients.

Parameter	Value
**Age, Mean (SD)**	52 (10)
**Gender: *n* (%)**	
male	58 (44)
female	74 (56)
BMI, mean (SD)	25.7 (4.1)
**Fibrosis staging: *n* (%)**	
F0–F2	64 (54.2)
F3–F4	54 (45.8)
**Activity grading: *n* (%)**	
A0–A1	28 (23.7)
A2–A3	90 (76.3)
HCV RNA × 103 IU/mL, median (Q25–Q75)	723 (197–2161)
**Sustained viral response (SVR), *n* (%)**	
positive	44 (42.7)
negative	59 (57.3)
**Genotype IL28B rs12979860, *n* (%)**	
CC	35 (26.5)
CT	74 (56.1)
TT	23 (17.4)
minor allele frequency	0.4545
**Genotype PNPLA3 rs738409, *n* (%)**	
CC	69 (52.3)
CG	58 (43.9)
GG	5 (3.8)
minor allele frequency	0.2576

**Table 2:** Distribution of diplotypes according to rs12979860 and rs738409 in the studied population.

Diplotype No.	rs12979860	rs738409	No. (%) of Cases with Shown Diplotype
1	CC	CC	22 (16.7)
2	CC	CG	13 (9.8)
3	CC	GG	0
4	CT	CC	39 (29.5)
5	CT	CG	31 (23.5)
6	CT	GG	4 (3.0)
7	TT	CC	8 (6.1)
8	TT	CG	14 (10.6)
9	TT	GG	1 (0.8)

The melting temperature (Tm) difference between the PCR products of the two primer pairs on clinical samples was at least 9 °C. Given the excellent primer performance in singleplex assays and the good separation of amplicon-melting domains, we decided to include our HRM primers in a duplex assay.

### 2.2. Characterization of Duplex HRM Assay

All samples reached a plateau before the end of the duplex real-time PCR amplification stage. The entire duration of amplification and acquisition protocol was less than 70 min. The melting peaks of rs12979860 amplicons were: 84.39 °C (CI95: 84.35–84.42 °C), 83.59 °C (CI95: 83.56–83.61 °C), and 83.79 °C (CI95: 83.77–83.81 °C), for the CC, TT, and CT genotypes, respectively. These values are very close to those found in singleplex reactions (Tm_duplex_ − Tm_simplex_ = −0.08 °C, *p* < 0.001, *t-*test). The rs738409 amplicons showed melting temperatures of 75.40 °C (CI95: 75.38–75.42 °C), 75.78 °C (CI95: 75.73–75.83 °C), and 75.34 °C (CI95: 75.33–75.36 °C), for the CC, GG, and CG genotypes, respectively, slightly higher than those observed in singleplex assays (Tm_duplex_ − Tm_simplex_ = 0.64 °C, *p* < 0.001). The melting temperature domains for the two SNPs did not overlap and could be analyzed separately in the instrument software. Normalization intervals were 66.5–69 and 78–79.5 °C for the rs738409 amplicon, 78.5–80 and 87–89 °C for the rs12979860 amplicon. RFU difference was used as a basis for melting curve clustering. The reproducibility of genotype call, tested in both intra- and inter-run settings, was 100%.

Genotype assignment for all clinical samples by duplex HRM was concordant with results of singleplex HRM testing, allele-specific PCR and Sanger sequencing. Genotyping accuracy for each SNP was 100% (95% CI: 0.9724–1), yielding 100% analytical sensitivity and specificity.

Two independent loci were assessed in each sample, making for a total of 264 genotypes interrogated in our patient group. From these, 263 were identified upon the first testing, resulting in a call rate of 99.62%. One sample showed an aberrant melting profile for rs738409, readily observable on visual inspection. Upon re-testing, a valid profile was obtained and the correct genotype was identified (CG).

The relatively wide range of genomic DNA concentrations (range: 0.5–73 ng/µL, median: 15 ng/µL; range concordant with qPCR Cq values) and sample quality (260/280 ratio: range 1.295–2.273, mean: 1.802, SD: 0.165) did not impair correct genotype identification. Call efficiency was not correlated with sample concentration or 260/280 ratio (*p* > 0.05, Spearman’s rank correlation test).

**Figure 1:**
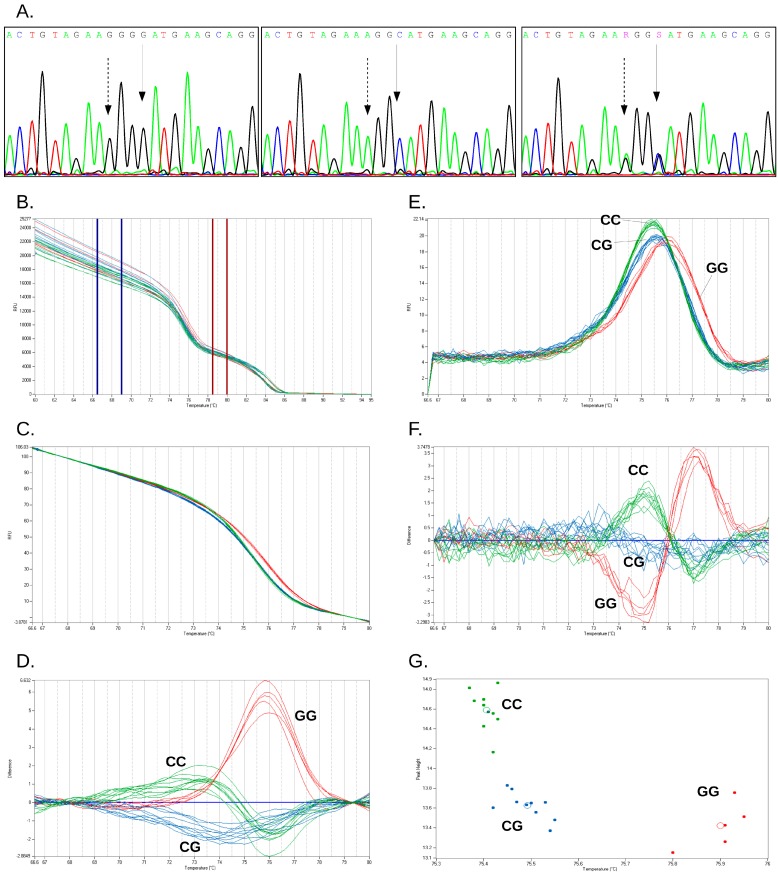
Genotyping of *PNPLA3* rs738409 SNP by sequencing and high-resolution melting curve analysis. (**A**) Representative Sanger sequencing traces for the three genotypes of rs738409 (from left to right: CC, GG, and CG; note that Sanger sequencing was performed in genome orientation, which is opposite to the rs); continuous arrows show rs738409 position; dashed arrows: rs738408 position; (**B**–**G**) Representative curves for high resolution melting of rs738409 amplicons: (**B**) non-normalized melting curves and placement of normalization intervals; (**C**) normalized melting curves; (**D**) RFU difference clustering; (**E**) negative first derivative of the normalized melting curves; (**F**) dF/dT difference clustering; and (**G**) scatter-plot. *PNPLA3* genotypes: red, GG; green, CC, blue, CG.

**Figure 2:**
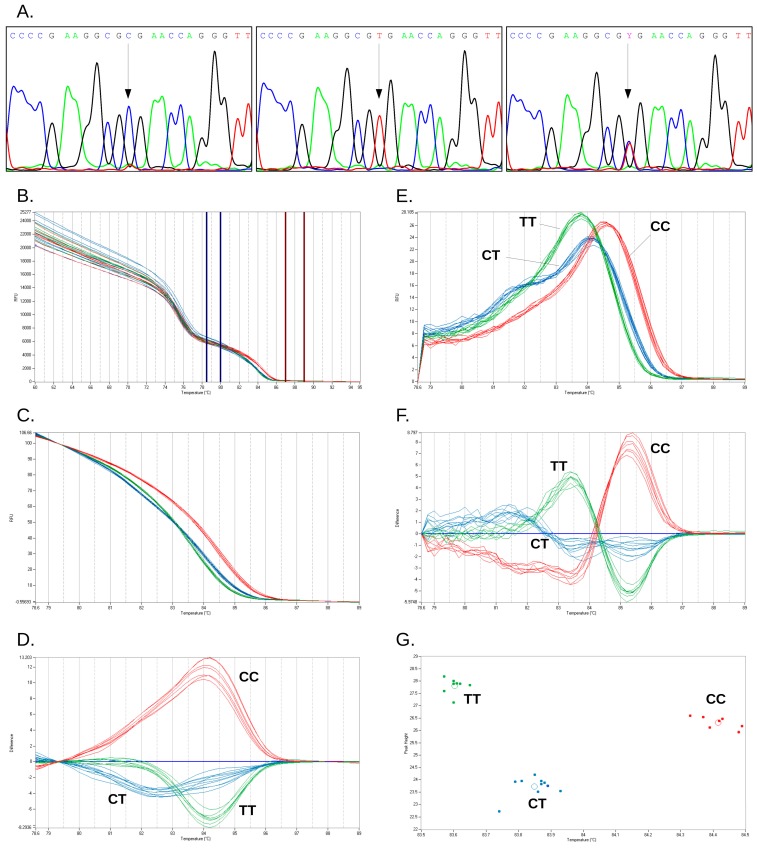
Genotyping of *IL28B* rs12979860 SNP by sequencing and high-resolution melting curve analysis. (**A**) Representative Sanger sequencing traces for the three genotypes of rs12979860 (from left to right: CC, TT, and CT); continuous arrows show SNP position; (**B**–**G**) Representative curves for high resolution melting of rs12979860 amplicons: (**B**) non-normalized melting curves and placement of normalization intervals; (**C**) normalized melting curves; (**D**) RFU difference clustering; (**E**) negative first derivative of the normalized melting curves; (**F**) dF/dT difference clustering; and (**G**) scatter-plot. *IL28B* genotypes: red, CC; green, TT; blue: CT.

Since the instrument software offers three different algorithms for HRM data analysis and clustering, we tested the influence of algorithm choice on genotype call. For rs12979860 genotype, all three analysis algorithms yielded 100% concordant results for the 132 clinical samples. Call efficiency was higher for the scatter-plot than for RFU difference and dF/dT difference algorithms (*p* < 0.0001, Dunn/Friedman test with post-test). For rs738409, both the RFU difference and dF/dT difference algorithms correctly genotyped all samples, with similar efficiencies (*p* > 0.05, Wilcoxon signed-rank test); the scatter-plot algorithm misclassified two samples out of 132. Representative results for the genotyping of rs738409 and rs12979860 by sequencing and the three available algorithms applied to duplex HRM are presented in Figure 1 and Figure 2.

**Figure 3:**
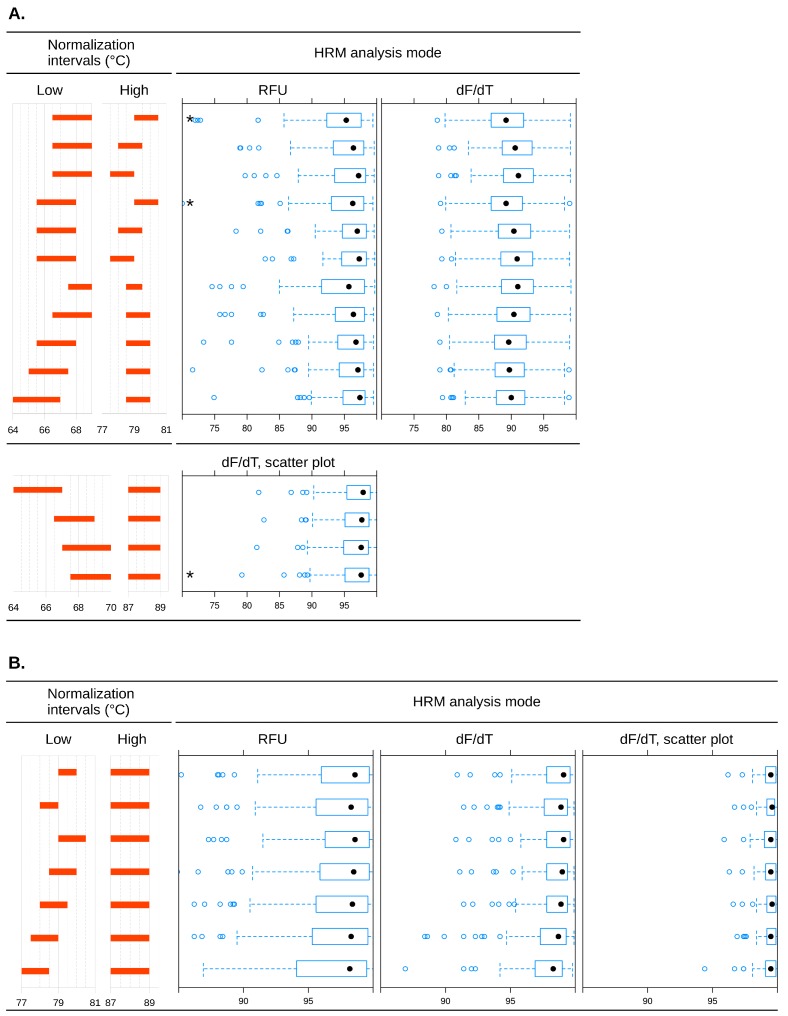
Influence of normalization temperature intervals and analysis mode on call efficiency in genotyping of rs738409 (**A**) and rs12979860 (**B**) by duplex real-time PCR and high resolution melting of amplicons. In combinations marked with an asterisk (*), call accuracy was >98%. For all other combinations, call accuracy was 100%. Filled circles (●): median genotyping efficiencies; open circles (○): possible statistical outliers.

It is known that the choice of normalization intervals for the pre-melt and post-melt domains may influence the results of HRM analysis [32]. To test the robustness of our duplex assay against changes in these parameters, we analyzed a subset of 70 clinical samples with all three available algorithms and a range of normalization intervals for each SNP. A relatively wide variation in the position and width of normalization intervals had minimal effects on both call efficiency and genotyping accuracy, regardless of analysis algorithm (Figure 3).

Various models of HRM analysis instruments are known to differ in their ability to perform SNP genotyping, especially in distinguishing between homozygotes [33]. We thus verified the performance of the duplex assay using a LightCycler Nano (Roche Diagnostics, Mannheim, Germany) as a different platform. The only adjustment of the analysis was the change of the lower temperature normalization interval for rs738409, from 66.5–69 to 69–71 °C. The intra-run reproducibility, tested on eight replicates of three samples, as well as the inter-run reproducibility, tested on 16 samples in 3 different days, were 100%. We additionally tested 57 samples (rs12979860: 13 CC, 36 CT, eight TT; rs738409: 33 CC, 22 CG, two GG) and all results were concordant with those obtained using the PikoReal 96 instrument and sequencing. The LightCycler Nano has two 16-well thermal blocks. We observed a temperature difference of 0.2 °C between the two blocks during the melting stage of the analysis. Although this difference did not affect genotyping in this case, we recommend that HRM analysis on the LightCycler Nano instrument be performed with melting standards on each block and samples be analyzed separately for the two blocks.

### 2.3. Discussions

Discovery of genetic polymorphisms associated with different outcomes in the natural history of disease, and with response to therapy is an important step forward in the personalized management of CHC patients.

*PNPLA3* polymorphism rs738409 was recently found to be associated with higher susceptibility to advanced forms of liver injury. In a meta-analysis including over 9900 patients, Singal *et al.* [19] addressed the effect of *PNPLA3* rs738409 genotype on the severity of liver fibrosis. They found that genotype G was significantly associated with advanced fibrosis in CHC patients, as well as in other etiologies of liver disease. The effect was similar between studies performed in Europe and the United States. In Caucasian CHC patients from Belgium, Germany and France, rs738409 G homozygosity was associated with advanced fibrosis stage and progression, as well as with steatosis [12]. *PNPLA3* genotype was also related to fibrosis progression in a complex study including, Swiss, French, American, and Australian Caucasian CHC patients [34]. Carriage of the G allele in *PNPLA3* was associated with an increased risk of steatosis in Swiss patients infected with HCV genotype non-3 [11]. A large Italian study found the rs738409 G allele to be independently associated with steatosis, fibrosis stage, and cirrhosis, treatment response and HCC development in CHC patients [13]. Similar results were found in Japanese patients [14]. Recently, an interaction between *PNPLA3* and *IL28B* genotypes in the development of steatosis has been described [35,36]. Moreover, the two loci seem to contribute to the prediction of clinical deterioration of HCV-related cirrhosis, defined as development of ascites, encephalopahy, variceal hemorrhage, HCC or liver-related death [37].

The development of direct acting antivirals (DAAs) has the potential to greatly improve the cure rate in CHC patients [38]. The ease of administration, high effectiveness, and low rate of side effects of new treatment regimens promise to change CHC from a disease that required highly specialized, but often unsatisfactory, treatment to a condition that may be easily managed by a general practitioner [39]. However, that promise remains distant at this time, since the predicted costs of the new antivirals are “as breathtaking as their effectiveness” [39]. Treatments including DAAs are about three times more expensive than PEG-IFN/RBV alone regimens and the cost per SVR achieved is also double [40]. Although cost-effectiveness analyses of new therapies show their advantages by decreasing long-term costs related to CHC-related morbidity [41,42], their wide use implies up-front costs that cannot be supported by medical systems in many regions [43], including Eastern European countries [44] such as Romania, where CHC treatment still relies on PEG-IFN/RBV even after several years since the introduction of the first DAAs.

Results from clinical studies suggest that innate immunity and endogenous IFN release may still be involved in viral clearance even in the new IFN-free regimens of CHC treatment. It was suggested that pre-treatment genotyping of *IL28B* may be useful in the case of these medications, although to a lower extent than in the classic combiation therapy [44]. Carriers of the favorable *IL28B* genotype may benefit from shorter treatment durations [44] or have high chances of response to conventional therapy, which implies significant cost reductions.

The duplex HRM assay developed by us is intended to facilitate the choice of effective cost-efficient therapy and follow-up schemes for HCV patients, especially in regions of the globe where the wide adoption of highly efficient IFN-free regimens is delayed. First, it may aid in the prediction of response therapy, especially in IFN-based schemes. Second, genetic testing may contribute to risk assessment in cases where treatment onset is deferred, either due to low chances of success of available classical therapy or to personal preferences of the patients who may wish to wait for the more clinically convenient oral therapies. In certain cases, predisposition to disease progression and the risk of complications may outweigh the benefits of newest medications if predicted the waiting time is long [45].

To our knowledge, this is the first report on a diagnostic assay for the simultaneous genotyping of rs12979860 and rs738409, two independent factors associated with disease outcome in CHC.

After verifying their proper performance in separate reactions, we included the HRM primers in a duplex format. Due to differences in amplification efficiencies, we empirically adjusted primer concentrations to obtain comparable melting heights for the competing amplicons. We observed that a larger melting peak for the rs738409 than for the rs12979860 amplicon improved genotyping of the former SNP, probably due to the lower Tm difference between the homozygote amplicons in the case of rs738409. The best results were obtained with final concentrations of 300 and 800 nM for rs12979860 and rs738409 HRM primers. The higher concentration of rs738409 HRM primers may be responsible for the increased Tm of specific amplicons in the duplex *vs.* simplex reactions, possibly through a higher amount of PCR product [46]. We, thus, recommend that, when designing multiplex assays based on melting curve analysis, one should consider enough distance between melting domains to allow for Tm variations linked with primer concentration optimization.

According to current recommendations on analytical validation of laboratory diagnostic procedures (*i.e*., ISO 15189), we examined the influence of common variables (concentration and quality of DNA samples, melting curve normalization intervals, HRM analysis algorithm, and occurrence of other sequence variations in the vicinity of targeted SNPs) on genotype identification and determined our assay as highly robust (100% accuracy for each SNP, 100% analytical sensitivity and specificity).

It is known that sequence similarity of several neighboring regions may negatively influence genotyping of *IL28B* SNPs [47]. A PrimerBLAST search suggested that our HRM primers for rs12979860 may be prone to amplify a second fragment of the same size upstream of the intended target. However, the reverse primer has three mismatches to this second genomic region, which is expected to severely impair its non-specific hybridization [48]. In addition, we employed touch-down PCR to prevent non-specific amplification in the *IL28B* region. The full concordance of the HRM results with those of allele-specific PCR and direct sequencing shows that our HRM primers are specific for the genotyping of rs12979860.

**Figure 4:**
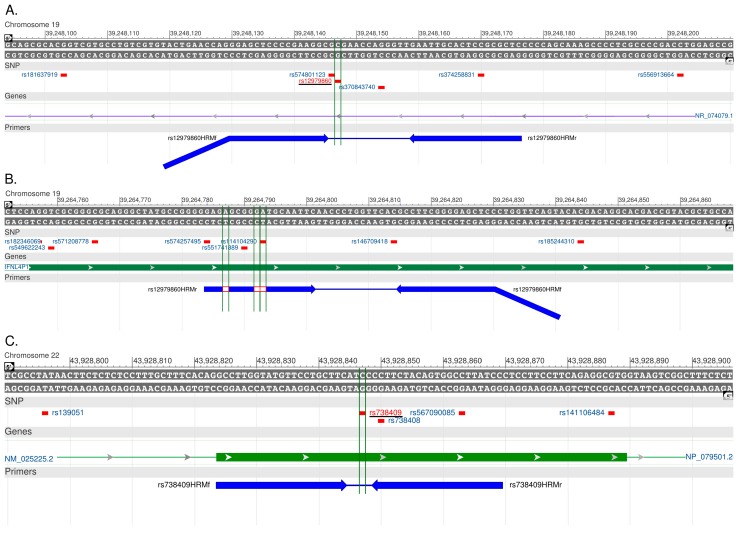
Positions of high-resolution melting analysis primers relative to human genome. (**A**,**C**) Specific binding positions of rs12979860 and rs738409 HRM primers, respectively; (**B**) Partial complementarity of rs12979860 HRM primers with an unintended target sequence in the *IL28B* region. Primers are shown in solid arrows. The added 5′ tail of the reverse rs12979860 HRM primer is shown as a diagonal solid line. Mismatches of the forward rs12979860 HRM primer to the genomic sequence are shown in open rectangles. SNPs in the respective regions are shown. Target variants are underlined.

Two other SNPs (Figure 4) inventoried in the dbSNP (as of 12 January 2015) may occur in the region amplified by the rs12979860 HRM primers and may affect the melting curve profile: rs574801123 A/G (minor allele frequency, MAF = 0.0006) and rs370843740 G/T (MAF not known). These variations are very rare in the general population and were not found in our cohort. However, their presence is expected to be readily detectable by HRM; in this case, samples can be referred to sequencing for proper genotyping. On the contrary, probe-based genotyping methods may be negatively influenced by these variants due to their close proximity to rs12979860 (1 and 7 bp distance, respectively). The amplicon produced with our rs738409 HRM primers is expected to include no other sequence variations besides rs738409.

A common SNP (rs738408 A/G) can occur at the 3′ penultimate nucleotide of the rs738409 HRM reverse primer binding site. We found genotypes GG, AA, and GA of rs738408 SNP in 55.3%, 41.5% and 3.3% of the sequenced samples, respectively. The presence of these variants did not impair correct genotyping of the target rs738409 in either simplex or duplex assays. Two other SNPs in the binding sites of our HRM reverse primers (Figure 4), rs374258831 C/T (MAF not known) and rs567090085 C/T (MAF = 0.0002), are also not expected to affect the performance of the assay, due to their internal positions relative to primers [48]. These variations were not found in our patients.

We finally verified the performance of the assay on a different HRM platform. For that purpose we chose LightCycler Nano, an instrument within the same price range as PikoReal 96, but presenting several differences in comparison to it: the thermal block (two blocks with 16 low profile wells of 100 µL *vs.* one block with 96 wells of 20 µL), optical component design, fluorescence acquisition rate (20 *vs.* five acquisitions per °C) and mode (12 channels simultaneously *vs.* one channel). Despite these differences, our assay performed similarly on both instruments. A recent technical comparison [49] shows that other HRM instruments on the market perform at least as well as the PikoReal 96 in genotyping amplicons in the length range of 50–100 bp, similar to those of our duplex test. We thus expect that our assay also performs well on other platforms.

Single-tube simultaneous genotyping of non-related SNPs may improve throughput and turnaround times in molecular diagnostic laboratories. The method described herein is less expensive than probe-based assays and definitely faster than Sanger sequencing, without sacrifices in analytical performance. Omission of the sample concentration normalization step does not seem to affect the assay accuracy. It further improves processing times and reduces the use of consumables. The closed-tube format, excluding further manipulations of the PCR products, also limits the contamination risks implied by other methods such as sequencing, gel electrophoresis and hybridization assays.

## 3. Experimental Section

### 3.1. Patients and Samples

The HRM assay was validated on samples from 132 Romanian patients with chronic HCV genotype 1 infection (Table 1). Information on liver fibrosis stage, assessed by liver biopsy or established non-invasive markers (FibroMax/ Fibrotest) prior to treatment onset, was available for 118 patients. A subset of 103 patients completed antiviral treatment with PEG-IFN and ribavirin between 2011 and 2014. The study was approved by the institutional Ethics Committee and all participants signed an informed consent form for inclusion in the study. Genomic DNA was purified from whole blood on K3EDTA or buffy coat samples using a spin column method (High Pure PCR Template Preparation Kit, Roche Diagnostics, Mannheim, Germany). The concentration and quality of the DNA samples were assessed by spectrophotometry, through the measurement of absorbance at 260 nm and the absorbance ratio at 260 and 280 nm, respectively. Concentration normalization of clinical samples prior to real-time PCR and HRM analysis was not performed.

### 3.2. Primer Design

HRM primers were designed with the online uDesign^SM^ application (www.dna.utah.edu/udesign/index.html) [50]. Their alignment with the genomic reference sequence is shown in Figure 4. A 5′ tail was added to the rs12979860 forward HRM primer for a better separation of the melting temperature domains of the rs12979860 and rs738409 amplicons. Allele-specific, cloning and sequencing primers were designed using Primer3 software [51] and verified for specificity with Primer-BLAST [52]. OligoAnalyzer 3.0 (https://eu.idtdna.com/calc/analyzer) [53] was used to assess the propensity of primers to form loops or dimers. In the case of allele-specific primers, a nucleotide mismatch was introduced in the penultimate 3′ position to increase specificity, and a GC tail was added to the 5′ end of one primer per SNP to facilitate visual discrimination of the amplicons by melting curve.

### 3.3. Construction of Melting Standards

Standard templates corresponding to each allele of the rs12979860 and rs738409 sites were generated from human genomic DNA by site-directed mutagenesis via overlap-extension PCR, employing primers listed in Table 3 and Phusion HS Polymerase (Thermo Fisher Scientific, Vantaa, Finland), according to the manufacturer’s instructions. Template point mutations were confirmed by sequencing. Templates corresponding to each allele were used as homozygote standards. Heterozygote templates were prepared by mixing homozygote standards in equimolar concentrations. Melting standards were adjusted to concentrations yielding quantification cycles (Cq) similar to purified clinical samples.

### 3.4. Real-Time PCR and High-Resolution Melting Analysis

Real-time PCR and melting experiments were performed in white plates on the PikoReal 96 (Thermo Fisher Scientific, Vantaa, Finland) instrument or in 100 µL low profile clear tubes on the LightCycler Nano (Roche Diagnostics, Mannheim, Germany), with 10 µL reaction volumes. Default temperature ramp rates (7 °C/s, as indicated by the instrument software) were used throughout the study.

Allele-specific real-time PCR mixture contained 2 µL DNA, 5 µL of Universal FastStart SYBR Green master mix with ROX (Roche Diagnostics, Mannheim, Germany), and primers at a final concentration of 400 nM for rs12979860 and 200 nM for rs738409. The temperature steps were 95 °C for 10 min., followed by seven cycles of 95 °C for 10 s and 67 °C for 30 s, with 0.5 °C decrease at each cycle, then 45 cycles at 95 °C for 10 s and 63 °C for 25 s. The final melting protocol was performed from 60 to 95 °C, with fluorescence readings every 0.2 °C on the PikoReal 96 or every 0.05 °C on the LightCycler Nano.

For HRM analysis, 2 µL DNA were amplified with the 2× SensiFast HRM master mix (Bioline, London, UK). Simplex reactions were performed with HRM primers for rs12979860 and rs738409 at final concentrations of 400 and 200 nM, respectively. The thermal protocol started with enzyme activation at 95 °C for 3 min, followed by seven cycles at 95 °C for 10 s and 67 °C for 30 s, with 0.5 °C decrease at each cycle, then 40 cycles at 95 °C for 10 s, and 63 °C (for rs12979860) or 60 °C (for rs738409) for 25 s.

**Table 3:** Primer sequences for HRM assays, allele-specific PCR, Sanger sequencing, and cloning.

Primer Name *^a^*	Nucleotide Sequence (5′→3′) *^b^*	Amplicon Length (bp)	Corresponding Human Genomic Region *^c^*
rs12979860HRMf	*CGAGGCGACCAC*GGAGCTCCCCGAAGGC	-	39248130–39248145
rs12979860HRMr	GAGCGCGGAGTGCAATTC	59	39248176–39248159
rs12979860aspf	TCTGCACAGTCTGGGATTCC	-	39248055–39248074
rs12979860aspCr	*CGGCGGGGCGGCC*GAGTGCAATTCAACCCTGGTT*G***G**	128	39248169–39248147
rs12979860aspTr	GAGTGCAATTCAACCCTGGTT*G***A**	115	39248169–39248147
rs12979860Seqf	*CATAGCATTTTTATCC*ACCTCTGCACAGTCTGGGAT	-	39248052–39248071
rs12979860Seqr	*CAAATTGTGAATTC*GCGCGGAGTGCAATTCAAC	153	39248174–39248156
rs12979860cf	GGACGAGAGGGCGTTAGAG	-	39247858–39247876
rs12979860cr	GTGCACGGTGATCGCAGAAG	675	39248532–39248513
rs12979860mCr	CAACCCTGGTTC**G**CGCCTTC	302	39248159–39248140
rs12979860mTr	CAATTCAACCCTGGTTC**A**CGCCTTC	307	39248164–39248140
rs12979860mCf	GAAGG**C**GCGAACCAGGGTTG	393	39248140–39248159
rs12979860mTf	GAAGGCG**T**GAACCAGGGTTGAATTG	393	39248140–39248164
rs738409HRMf	GCCTTGGTATGTTCCTGCTTC	-	43928824–43928844
rs738409HRMr	GGATAAGGCCACTGTAGAAGG	46	43928869–43928849
rs738409aspGf	*CGCGGCGGCC*CCTTGGTATGTTCCTGCTTCA*C***G**	97	43928825–43928847
rs738409aspCf	CCTTGGTATGTTCCTGCTTCA*A***C**	87	43928825–43928847
rs738409aspr	CTAGCAGAGAAAGCCGACTTAC	-	43928911–43928890
rs738409Seqf	*ACTGAGCGAATTC*TTGCTTTCACAGGCCTTGG	-	43928812–43928830
rs738409Seqr	*CATGCTGGAATTC*CGCTAGCAGAGAAAGCCGAC	128	43928913–43928894
rs738409cf	CACCGATCTAGCCCCTTTCA	-	43928498–43928517
rs738409cr	TAAGTTTTGCTGCCCGGGTA	583	43929080–43929061
rs738409mGr	CTGTAGAAGGG**C**ATGAAGCAGGAAC	361	43928858–43928834
rs738409mCr	CTGTAGAAGGG**G**ATGAAGCAGGAAC	361	43928858–43928834
rs738409mGf	GTTCCTGCTTCAT**G**CCCTTCTACAG	247	43928834–43928858
rs738409mCf	GTTCCTGCTTCAT**C**CCCTTCTACAG	247	43928834–43928858

*^a^* Abbreviations used in primer names: HRM, primers used in high resolution melting assays; asp, allele-specific primer; Seq, sequencing primer; m, primer used in directed mutagenesis by overlap-extension PCR; c, cloning primer (external); *^b^* Underlined sequences in *Italic* are not complementary to the genomic reference sequence; letters in boldface designate variant-specific nucleotides; *^c^* NC_000019.10 for rs12979860 and NC_000022.11 for rs738409.

For duplex HRM analysis, rs12979860 and rs738409 primers were used at final concentrations of 300 and 800 nM, respectively. The amplification step included incubation at 95 °C for 3 min, followed by seven cycles at 95 °C for 10 s and 67 °C for 30 s, with a decrease in the aligning temperature of 0.5 °C/cycle, then 40 cycles at 95 °C for 10 s and 60 °C for 25 s. Standard templates, positive controls consisting of DNA samples with known genotypes, and PCR-grade water as no template control were tested in all analytical series, along with clinical samples.

High resolution melting was preceded by amplicon denaturation at 95 °C for 1 min and heteroduplex formation at 40 °C. Reaction products were then gradually heated from 60 to 95 °C in steps of 0.2 °C per 4 s, with fluorescence readings at every step. The PikoReal instrument software (ver. 2.2, Aug 2013, Thermo Fisher Scientific, Vantaa, Finland) is able to assign a call to each melting curve, using a hierarchical clustering algorithm and input calls of standard melting templates. First, fluorescence values are normalized according to user-adjustable pre-melting and post-melting temperature intervals, wherein the fluorescence of each curve is considered 100% and 0% respectively. Then, fluorescence curves can be transformed by three different methods to facilitate clustering. The first method (RFU difference) relies on the calculation of the difference between fluorescence of all samples and a reference calculated automatically. In the second (dF/dT difference), the difference between the (negatively transformed) first derivative of the fluorescence of each sample and that of a reference is calculated. The third method only considers the melting peaks of the normalized curves, resulting in a scatter plot with temperature and peak height as coordinates.

The LightCycler Nano software (ver. 1.0, June 2011, Roche Diagnostics, Mannheim, Germany) only supports the relative fluorescence difference algorithm and is not able to automatically assign genotypes if more than one melting domain is present in one sample, as in a duplex assay. Genotypes were thus assigned manually by two users, blinded to the initial results from the PikoReal 96 instrument.

### 3.5. Reproducibility

Intra-run reproducibility of the duplex assay was assessed on the PikoReal 96 using representative samples for all nine possible diplotypes by testing ten replicates on one reaction plate. Except for the rs12979860/rs738409 CC/GG diplotype, for which a mixture of standard templates was used, available clinical samples were used for the other eight diplotypes. Inter-run reproducibility also covered all diplotypes and was evaluated by testing 22 clinical samples and three combinations of standard templates (CC/GG, TT/CC, CT/GC for rs12979860/rs738409), in three different days, by two users. Standard templates were used for CC/GG, and a single clinical sample was used for TT/GG. For each of the seven remaining diplotypes, three clinical samples were tested in each run.

Reproducibility was verified on the LightCycler Nano for all genotypes. In the intra-run setting, three samples (rs12979860/rs738409: CC/CG, CT/GG, TT/CC) were tested in eight replicates, whereas in the inter-run assessment, 16 samples (rs12979860: six CC, five CT, five TT; rs738409: eight CC, five CG, three GG) were tested on three different days.

### 3.6. Sanger Sequencing

A subgroup of 123 samples, for which DNA was available, covering all genotypes, and including samples used in reproducibility assessment, were verified by direct sequencing. Briefly, 10 ng of each sample DNA was amplified on an Applied Biosystems 9700 thermal cycler using the sequencing primers listed in Table 3 and Phire II DNA polymerase (Thermo Fisher Scientific, Vantaa, Finland), as per manufacturer’s instructions. PCR products were spin-column purified (DNA Clean and Concentrator 5 kit, Zymo Research, Irvine, CA, USA), verified and quantified by agarose gel electrophoresis. Amplicons were concentration-normalized and sent to BaseClear (Leiden, The Netherlands) for Sanger sequencing.

### 3.7. Statistical Analysis

Genotype frequencies were assessed using software [54,55] available from the Online Encyclopedia for Genetic Epidemiology studies (http://oege.org/sofrware) [56]. Other statistical calculations employed two-sided parametric or non-parametric tests, as required, and are mentioned in the Results section. Statistical significance threshold was set at 0.05.

## 4. Conclusions

Our assay offers a rapid, accurate and affordable means for the simultaneous genotyping of *IL28B* rs12979860 and *PNPLA3* rs738409 polymorphisms, useful in the personalized management of patients with CHC.
